# The Involvement of Lactosylceramide in Central Nervous System Inflammation Related to Neurodegenerative Disease

**DOI:** 10.3389/fnagi.2021.691230

**Published:** 2021-07-19

**Authors:** Wen Yu, Jun Ying, Xifeng Wang, Xing Liu, Tiancheng Zhao, Sungtae Yoon, Qingcui Zheng, Yang Fang, Danying Yang, Fuzhou Hua

**Affiliations:** ^1^Department of Anesthesiology, The Second Affiliated Hospital of Nanchang University, Nanchang, China; ^2^Key Laboratory of Anesthesiology of Jiangxi Province, Nanchang, China; ^3^Department of Anesthesiology, The First Affiliated Hospital of Nanchang University, Nanchang, China; ^4^Mailman School of Public Health, Columbia University, New York, NY, United States; ^5^Helping Minds International Charitable Foundation, New York, NY, United States

**Keywords:** neurodegenerative diseases, glycosphingolipid, lactosylceramide, gangliosides, lipid raft, membrane microdomain

## Abstract

Neurodegenerative diseases are a class of slow-progressing terminal illnesses characterized by neuronal lesions, such as multiple sclerosis [MS, Alzheimer’s disease (AD), Parkinson’s disease (PD), and amyotrophic lateral sclerosis (ALS)]. Their incidence increases with age, and the associated burden on families and society will become increasingly more prominent with aging of the general population. In recent years, there is growing studies have shown that lactosylceramide (LacCer) plays a crucial role in the progression of neurodegeneration, although these diseases have different pathogenic mechanisms and etiological characteristics. Based on latest research progress, this study expounds the pathogenic role of LacCer in driving central nervous system (CNS) inflammation, as well as the role of membrane microstructure domain (lipid rafts) and metabolite gangliosides, and discusses in detail their links with the pathogenesis of neurodegenerative diseases, with a view to providing new strategies and ideas for the study of pathological mechanisms and drug development for neurodegenerative diseases in the future.

## Introduction

Lactosylceramide is an important intermediate in the synthesis of sphingolipids. As a biologically active sphingolipid, it can directly participate in the conduction of signals between cells and can also be decomposed in ganglia with important effects in the body with the involvement of biologically active enzymes, glycosides, and lipid rafts (Chatterjee and Pandey, 2008; Chatterjee et al., 2021). Lactosylceramide (LacCer) is synthesized by the transfer of glucose and galactose from UDP-glucose and UDP-galactose to ceramide by galactosylceramide synthetase. The synthesis and decomposition of LacCer are complicated. On one hand, there is controversy about the source of the initial skeleton of ceramide in the synthesis of LacCer. Previous studies have suggested that ceramide is mainly synthesized *de novo* on the endoplasmic reticulum (Gault et al., 2010). Some scholars have proposed hydrolysis with neutral and acidic sphingomyelinase (Smase) as the source ceramide. The produced ceramide may also be involved in the synthesis of the precursor of lactosylceramide. On the other hand, decomposition products of LacCer includes a variety of gangliosides, such as GM2, GM3, and GD1 (Wiegandt, 1995). Each class of gangliosides includes multiple subtypes, and they are involved in a variety of diseases, such as diabetes (Inokuchi, 2014), cancer (Groux-Degroote et al., 2018), and cardiovascular disease. LacCer promotes the accumulation of abnormal proteins (Srivastava et al., 2019), abnormal signaling (Filippatou et al., 2020), and neurotoxicity of gangliosides (Dhanushkodi et al., 2019), which leads to the occurrence and further progression of neurodegenerative diseases.

Gangliosides are an essential component of cellular lipid rafts, which are critical components of various brain cells, such as astrocytes, microglia, and neurons (Grassi et al., 2020). There have been many controversies over the existence of lipid rafts. As early as 2006, scholars have formed a unified definition of lipid rafts (Pike, 2006): “The membrane raft is small (10–200 nm), heterogeneous, and highly dynamic. The sterol and sphingolipid-rich domains separate cellular processes. Small rafts can sometimes be stabilized to form larger platforms through protein–protein and protein–lipid interactions.” Lipid rafts regulate cellular metabolism and the initiation of signaling by organizing pathway components into ordered micro-regions on the cell surface (Simons and Sampaio, 2011). Lipid rafts mediate changes in cell biological functions, and their dysfunction can promote the progression of related diseases (Michel and Bakovic, 2007). In neurodegenerative diseases, there is a correlation between the identification of cell lipid rafts and functional changes (Mesa-Herrera et al., 2019). Therefore, studies have also pointed out the inhibitory effect of LacCer upstream of lipid rafts. It is related to the promotion of the inhibition and recovery of lipid rafts. It cannot be ruled out that studies on the role of lipid rafts in neurodegenerative diseases may promote research on related pathological mechanisms and drug development.

There is a great variety of neurodegenerative diseases, but they are all characterized by damage to the structure and function of neurons in specific areas of the brain, which ultimately leads to neuron loss (Katsnelson et al., 2016). Most progressive diseases have a slow onset and irreversible loss of function, such as AD, PD, MS, and ALS. With the increasing aging and urbanization of the global population, the incidence of neurodegenerative diseases is increasing year by year globally, especially in developing countries. The pathogenesis of neurodegenerative diseases is complex. Common explanations include the protein degeneration theory (Kurtishi et al., 2019), lipid metabolism theory (Hallett et al., 2019), and mitochondrial theory (Li et al., 2017), but the drugs developed based on these theories have not achieved satisfactory results. For example, AD is widely considered to be related to the abnormal accumulation of β amyloid in brain cells, but none of the drugs developed based on this theory have succeeded in clinical trials. Although some progress has been made in the development of drug for some neurodegenerative diseases such as MS, the present understanding of the underlying pathological mechanisms is not necessarily perfect. Some clinical data indicate that there are lipid abnormalities in the serum and cerebrospinal fluid of patients with neurodegenerative disease. At the same time, there are also experiments demonstrating that lipids, such as lactosylceramide, can participate in the development of neurodegeneration. Therefore, it is necessary to assess how LacCer might mediate the pathogenesis of neurodegenerative diseases.

### LacCer Induces Neurodegeneration by Activating Astrocytes Leading to Neuroinflammation

Astrocytes are neuroimmune cells and also the most abundant cells in the brain. Their main functions include supporting, nourishing, and creating a suitable microenvironment for neuron survival (Sofroniew and Vinters, 2010; Colombo and Farina, 2016). When astrocytes are activated to enter a pro-inflammatory state (Sofroniew, 2014), the produced pro-inflammatory factors, chemokines, reactive oxygen species, and secondary messengers work together to induce neuroinflammation, resulting in multifaceted dysfunction, including but not limited to abnormal autophagy (Bosch and Kielian, 2015; Thangaraj et al., 2020), lipid metabolism disorders (Gonzalez-Guevara et al., 2020), energy supply disorders (Trudler et al., 2015), and other abnormalities of cell physiology, which ultimately destroys neurons and synaptic function. At the same time, neuroinflammation hinders cell and tissue repair and regeneration, resulting in the irreversibility of neurodegeneration (Schain and Kreisl, 2017). Schain and Kreisl (2017) divided astrocytes into type A1 (a subtype of astrocytes with a neurotoxic phenotype) and type A2 (a subtype of astrocytes with neurotrophic and neuroprotective properties) (Schain and Kreisl, 2017). The degree and source of the injury determine which phenotype the astrocytes express. Lipids play an important role in neuroinflammation (Won et al., 2007; Jones and Ren, 2016). Disorders of lipid metabolism are related to the phenotypic expression of astrocytes, and LacCer is a crucial product of lipid metabolism. Recent studies have shown that LacCer activates astrocytes through various pathways, such as the B4GALT6-LacCer axis (Mayo et al., 2014), cPLA2-MAVS axis (Chao et al., 2019), as well as the Ras/ERK1/2 and IκB/NF-κB pathways (Pannu et al., 2005). This activation promotes the release of pro-inflammatory factors, the infiltration of the CNS by peripheral leukocytes and other inflammatory changes, which further causes neurodegeneration.

#### The Axis of B4GALT6-LacCer

LacCer synthase is the key enzyme that synthesizes lactosylceramide by transferring galactose from uridine diphosphate (UDP)-galactose to glucosylceramide (GlcCer). Studies have found that B4galt may be responsible for LacCer synthesis (Lee et al., 2001; Kolmakova and Chatterjee, 2005). The B4galt family consists of seven genes, each of which plays a different role in the production of lipid metabolites. As early as 1998, the enzymeβ-1,4-galactophore glycosyltransferase 6(B4galt6) has purified from rat brain homogenate (Nomura et al., 1998). At the same time, an EAE mouse model was used to detect elevated levels of LacCer in the CNS, and the high expression of B4galt6 in astrocytes was confirmed by transcriptome sequencing and quantitative PCR. Consequently, it was inferred that B4galt6 is the essential regulatory gene for LacCer synthesis. Furthermore, Mayo studies confirmed the specific mechanism by which LacCer synthesized by B4galt6 is involved in the regulation of atrocity activation (Mayo et al., 2014). (1) LacCer under the influence of B4galt6 activates astrocytes *via* the NF-κB and IRF-1 pathways in an autocrine manner; (2) LacCer in astrocytes controls the recruitment and activation of microglia and CNS-infiltrating monocytes by regulating the production of chemokine CCL2 and granulocyte-macrophage colony-stimulating factor (GM-CSF) in a paracrine manner.

#### LacCer Activates the CPLA2-MAVS-NF-KB Pathway

Phospholipase A2 (PLA2) is a ubiquitous enzyme in eukaryotic organisms, either in the form of calcium-independent (iPLA2), cytoplasmic (CPLA), or secretory PLA2 (SPLA2) (Linkous and Yazlovitskaya, 2010). Under physiological conditions, phospholipid structure, lipid peroxidation state, calcium and/or G protein interactions regulate the activity of cPLA2. When cPLA2 is abnormally activated, it leads to membrane deacetylation, activation of the second messenger system, and uncontrolled calcium influx, which can cause severe apoptosis. It was found that CPLA2 plays a vital role in a variety of pathological processes, such as microbial infection, ARDS, airway inflammation, cancer, AD, and other diseases (Keane et al., 1997; Bonventre, 1999; Nagase et al., 2000; Nakanishi and Rosenberg, 2006). As an enzyme responsible for the decomposition of membrane phospholipids and phosphatidylcholine, activated CPLA2 is responsible for the production of arachidonic acid (AA) (Nakamura et al., 2010, 2013), which contributes mainly to the synthesis of pro-inflammatory lipid second messengers such as prostaglandins, leukotrienes, and alkanes (Noor et al., 2008). Researchers originally thought that C1P enhances the activation of cPLA2 by binding to its C2 domain (Stahelin et al., 2007). However, Rothhammer et al. (2018) and Chao et al. (2019) recently found that LacCer in astrocytes activates cPLA2, which interacts with the caspase activation and recruitment domain (CARD) domain of the mitochondrial antiviral signal protein (MAVS) to induce NF-κB-mediated pro-inflammatory transcription. At the same time, the binding of MAVS to hexokinase 2 (HK2) is inhibited, reducing the amount of lactic acid to support neuron survival. The mechanism by which the cPLA2-MAVS-NF-κB pathway drives astrocyte activity provides new potential strategies for the treatment of neurodegeneration.

#### LacCer-Ras/ERK1/2 and LacCer-IκB/NF-κb Pathways

**FIGURE 1:**
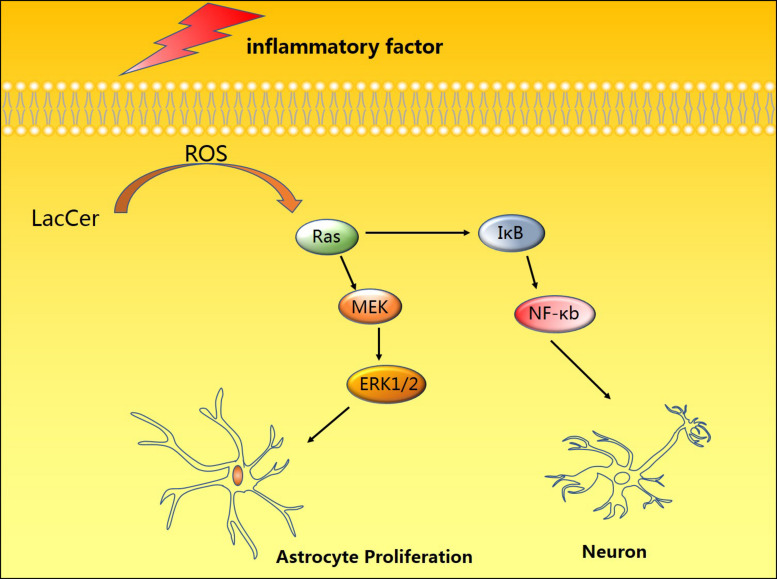
Induction by pro-inflammatory factors and toxic substances can promote the accumulation of LacCer in cells, thereby activating the Ras-MEK-ERK1/2 and IκB/NF-κB pathways to promote astrocyte proliferation and neuronal apoptosis.

In early studies, nitric oxide (NO) was considered an endothelium-dependent vasodilator involved in the regulation of vascular contraction (Pirahanchi et al., 2020). However, with the development of research, there is increasing evidence that NO may be involved in the occurrence and development of a variety of diseases, such as various studies indicating that NO-mediated inflammation is often indispensable for neurodegeneration (Dawson et al., 1993; Tewari et al., 2020). Lipopolysaccharide (LPS) is an important cell wall component of Gram-negative bacteria, which can effectively activate astrocytes to induce neuroinflammation in animal models of neurodegenerative diseases. Pannu et al. (2004) used a rat spinal cord injury (SCI) model to investigate the mechanism of LPS-mediated inflammation. They found that LacCer regulates gene expression of the inflammatory mediator iNOS through the Ras/ERK1/2 and IκB/NF-κB pathways (Figure 1). The unique advantage of PDMP in inhibiting LacCer-mediated neuroinflammation also offers strong evidence for clinical application (Mayo et al., 2014).

### LacCer Is Decomposed to Produce Gangliosides and Participates in the Construction of Lipid Rafts

**FIGURE 2:**
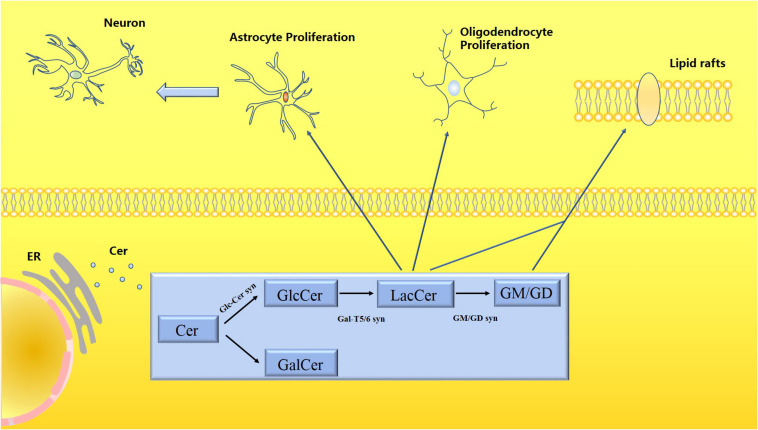
Schematic diagram of the metabolism of LacCer and gangliosides. Ceramide (Cer) is processed in the endoplasmic reticulum to produce glycosphingolipids (GSLs), which are divided into glucosylceramide (GlcCer) and galactose ceramide (GalCer). LacCer participates in the activation of astrocytes and microglia to trigger neuroinflammatory responses. Both LacCer and gangliosides participate in the formation of lipid rafts, which have a stable membrane structure, regulating material transportation and signal transmission.

Lactosylceramide is the central intermediate of the synthesis of complex sphingolipids and the starting point for the synthesis of all gangliosides. Glucose is transferred from LacCer to ceramide and forms gangliosides by combining with monosaccharides. Gangliosides are present in almost all vertebrate cells and tissues but are especially abundant in the brain. There are many types of gangliosides, among which GM1, GD1a, GD1b, and GT1b are the most common, and GM1 accounts for about 28% of the total human brain gangliosides (Schnaar et al., 2014). Studies have confirmed links between gangliosides and cancer signal transduction (Groux-Degroote et al., 2018), insulin resistance (Sasaki et al., 2019), cellular responses to infection (Rueda, 2007), and CNS inflammation (Furukawa et al., 2018). Ganglioside antibodies are expressed in large quantities in the CNS and play an important role in stress resistance of nerve tissue and promotion of tissue repair. Studies on mutant mice lacking gangliosides have found that when ganglioside genes are not expressed, CNS complement activation and neuroinflammation were found in almost all mice. When testing the serum of patients with dementia, 66% of 106 patients with dementia had IgM anti-GM1 antibodies, which were significantly related to the occurrence of dementia (Hatzifilippou et al., 2014). However, the anti-GD1b and GQ1b antibodies were also confirmed to be associated with dementia. The occurrence of dementia is calculated relative to the control group, but the detection rate of patients with dementia is lower than 50%, and even the anti-IgM and anti-GQ1b antibodies accounted for only 9% of the cases, so gangliosides are considered to be related to neurodegenerative diseases. Although there is a potential correlation, not all gangliosides are expressed abnormally. It also appears that the main abnormal gangliosides expressed in different neurodegenerative diseases are distinct. At the same time, inhibiting the expression of the B3galt4 mRNA involved in the synthesis of ganglioside GM1 in patients with PD reduced the expression level of GM1 in SK-N-SH cells and enhanced the sensitivity of cells to neurotoxic stimulation (Verma and Schneider, 2019). The lack of gangliosides also interferes with the stability of membrane proteins at the axon-glia junction, such as the axonal adhesion molecule Neurofascin155 (NF155) and myelin-associated glycoprotein (MAG), which in turn destroys the integrity of synapses. In mice with a knockout of neuraminidase 3 and 4 genes (Pan et al., 2017), ganglioside GM3 is stored in microglia. The level of GM1 in neuronal axons is significantly reduced, which mediates neuroinflammation and neurodegeneration. Gangliosides have various biological activities in the body. They are involved in the synthesis of abnormal proteins, abnormal accumulation of gangliosides, destruction of synaptic stability, interference with information transmission, and enhancement of sensitivity to stressors in the pathological process of degenerative diseases (Figure 2).

In the cell membrane, glycolipids, sphingolipids, and cholesterol can combine in certain proportions to form membrane micro-domains that can regulate cell metabolism and signal transduction, which are also called lipid rafts. Lipid rafts play an essential role in cell physiology, with roles in infectious diseases (Luo et al., 2008), neuroinflammation (Miller et al., 2020), pathological pain (Santha et al., 2020), and hematopoiesis (Alomari et al., 2019), and cancer (Mollinedo and Gajate, 2020). Lipid rafts are highly dynamic domains in the cell membrane that can freely combine with larger lipid raft platforms as needed. This fluidity enables them to exert their functions, such as signal transduction, material transport, and endocytosis between cells. Gangliosides play an indispensable role in maintaining the structural stability of lipid rafts. In transgenic mice lacking gangliosides (Ohmi et al., 2011), the lack of gangliosides GM2, GD2, and GD3 leads to lipid raft disorder and downregulated expression of lipid raft markers. Complement genes in the nervous system are upregulated, and astrocytes and microglia are activated. In sheep suffering from Gaucher’s disease (Hein et al., 2017), the ratio of lactose ceramide, ganglioside, cholesterol, etc., in the body is unbalanced, which destroys the structure of lipid rafts. Therefore, the instability of lipid rafts is closely related to the imbalance of the environment within the cell membrane and neurodegeneration. Similarly, amyloid fibrils bind to the cell membrane (Bucciantini et al., 2012), leading to the aggregation and migration of GM1 on the cell surface. The reorganization of the lipid raft structure and changes in homeostasis contributes to fibril-mediated cytotoxicity. Understanding the close relationship between lipid rafts, neuroinflammation, and amyloid misfolding will broaden the prospects for targeted therapy of neurodegenerative diseases.

Ceramide (Cer) is processed in the endoplasmic reticulum to produce glycosphingolipids (GSLs), which are divided into glucosylceramide (GlcCer) and galactose ceramide (GalCer). LacCer participates in the activation of astrocytes and microglia to trigger neuroinflammatory responses. Both LacCer and gangliosides participate in the formation of lipid rafts, which have a stable membrane structure, regulating material transportation, and signal transmission.

## The Relationship Between LacCer and MS

Multiple sclerosis a chronic demyelinating autoimmune disease (Nylander and Hafler, 2012) and is the most common non-traumatic CNS disease in young patients. In most patients with multiple sclerosis (MS), clinical features are divided into two stages (Reich et al., 2018). First, the disease goes through a phase of spontaneous remission of relapse (relapsing-remitting multiple sclerosis, RRMS). Subsequently, the process of chronic inflammation continually interferes with axonal homeostasis, and neurodegeneration is further advanced, thus entering the progressive stage (secondary progressive multiple sclerosis, SPMS). There is evidence that chronic activation of the innate immune system in the CNS is closely related to the pathological process of progressive MS (Lassmann et al., 2012; Rothhammer et al., 2018; Wheeler et al., 2019), but the specific pathological mechanism of progressive MS is not clear. Consequently, the clinical treatment of MS is mainly focused on RRMS (Hempel et al., 2017).

It has been reported that astrocytes play an active role in RRMS and MS stages of severe injury (Brosnan and Raine, 2013; Yi et al., 2019). Astrocytes promote tissue damage by upregulating lymphocytes but can also limit inflammation and promotes tissue repair. Astrocytes themselves will be seriously damaged because of inflammation, which leads to dual dysfunction of the astrocyte, and the pro-inflammatory process dominates the development of the disease. Therefore, the effect and magnitude of the astrocyte activation pathway in MS lesions have become the focus of research. In a chronic progressive model of experimental autoimmune encephalomyelitis (EAE) in mice (Mayo et al., 2014), it was found that the level of LacCer is increased, and that EAE is aggravated by injecting LacCer, while the use of b4galt56 inhibitors (PDMP) lowers CNS levels of LacCer and inhibits disease progression. The mechanisms through which LacCer contributes to MS include promoting the production of the chemokine CCL2, granulocyte-macrophage clustering stimulating factor (GM-CSF), iNOS, and activation of the NF-κB pathway. The overproduction of NO is associated with neuronal cell death and demyelination in MS. Some researchers have found that lipopolysaccharide or IFN can increase lactosylceramide levels and induce the expression of the iNOS gene. Inhibition of LacCer synthase (GalT-2, glycosyltransferase) could inhibit the expression of iNOS caused by LPS/IFN-γ, and the effects of exogenous LacCer could be reversed. The chemokine CCL2 plays an important role in EAE by recruiting pro-inflammatory mononuclear cells to the CNS and is one of the main proinflammatory molecules in LacCer-upregulated astrocytes. GM-CSF is essential for the development of EAE (El-Behi et al., 2011). The NF-κB pathway is crucial for neuroinflammation, and blocking NF-κB activation mediated by LacCer reduces the recruitment of lymphocytes and macrophages, significantly reducing the severity of the disease in EAE.

## The Relationship Between LacCer and AD

Alzheimer’s disease (AD) is a progressive disorder associated with memory problems and cognitive decline (Tanzi and Bertram, 2005). AD has the highest incidence rate of all neurodegenerative diseases, with close to 35 million patients with AD in the world. Moreover, it is estimated that the number of patients worldwide will exceed 80 million by 2040 (Martinez Martinez and Mielke, 2017). AD is a neurodegenerative disease closely related to aging. The focus of research is on discovering specific mechanisms that lead to changes in the microenvironment of the brain with age. Studies have shown that amyloid protein is a decisive factor for disease production and severity in AD. For a long time, the international community believed that the main pathological characteristic of AD is the excessive deposition of amyloid in the hippocampus, leading to the formation of plaques and tangles of hyperphosphorylated tau protein inside cells, resulting in synaptic dysfunction (Goedert and Spillantini, 2006; Sutovsky et al., 2014). Based on the amyloid hypothesis, reversing the abnormal accumulation of Aβ is the key to AD treatment. However, many attempts to treat AD with anti-amyloid drugs have failed to achieve the desired outcome. At the same time, the “amyloid cascade hypothesis,” as demonstrated by related autopsy and imaging studies, cannot fully explain the neuronal damage observed in AD (Fillit et al., 1991; Struble et al., 2010; Varnum and Ikezu, 2012; Calsolaro and Edison, 2016). However, many studies have found that the content of lipids in the brains of patients with AD is abnormal. Studies have found that glycosphingolipids play a crucial role in Aβ-induced cellular stress, and that lipid rafts are involved in mediating cellular inflammation and toxic signaling. Moreover, there is accumulation of membrane proteins together with Aβ, which suggests that lipid metabolism disorders may be an essential part of the pathological process of AD. Ceramider levels showed opposite changes in different stages of AD lesions. Ceramider levels increased significantly in the early stage but showed attenuation in the late stage. The Jana A study confirmed that neurons have higher sensitivity to death in a high ceramider environment. Removal of ceramider and inhibition of ceramider synthesis may, therefore, be a viable treatment strategy for AD.

Ganglioside is a glycosphingolipid derivative containing one or more sialic acid residues, and it plays an essential role in the behavior of the central nervous system (CNS). It is reported that ganglioside metabolism is related to the AD pathology (Yanagisawa, 2015), and changes in the ganglioside profile were observed in patients with AD (Yanagisawa, 2007; Matsuzaki et al., 2010). H4APPsw cells can replicate some of the essential characteristics of AD. Cell experiments have also shown that gangliosides are significantly increased in H4APPsw cells. An increasing number of research studies have been based on gangliosides because of the protective impact of gangliosides in the CNS. Also, the lipid rafts formed by gangliosides might have an essential role in the occurrence and development of AD. While studying the pathogenic mechanism of ganglioside-mediated AD, the critical aspect is that GM1 forms a substantial constituent of lipid rafts, and alterations in lipid rafts might be the potential pathogenic mechanism of AD. In the pathogenic mechanism of AD, a crucial point is that GM1 constitutes an important limb of the lipid rafts, and that the change in lipid rafts forms as a potential clinical risk of AD (Staneva et al., 2018). In clinical studies (Liu et al., 2015), the content of platelet lipid raft protein in elderly patients with AD has no differences, but the ganglioside GM1 content of lipid rafts from the platelet is immensely greater as compared with the control group. Therefore, potentially, GM1 is a diagnostic marker for AD. Recent studies (Ariga, 2017; Fukami et al., 2017) have been pointing out that the level of cholinergic neuron-specific gangliosides in patients with AD is crucially risen, highlighting that gangliosides might have a key part in protecting the function of cholinergic neurons. Moreover, it has also been explored by studies that amyloid proteins could develop Ca^2+^ channels in lipid rafts and destroy membrane stability; hence, lipid rafts and gangliosides might be important targets for AD treatment.

Even though evidence shows that AD is closely related to gangliosides in brain tissue, the underlying mechanism is still unclear. However, in the body, gangliosides are not always co-localized. Previous studies (Pernber et al., 2012) have found that the brain tissue retains the presence of ganglioside GM1 more; and that GM2 constitute the most common ganglioside in myelin, which might be a representation of the disorder of myelin structure in the gray matter. Temporal gray matter consists of various vertebral cells, and GM2 is present in the form of vesicle aggregation; mediating brain dysfunction. Concerning the distribution of varying types of gangliosides having the same type also differs largely. It has been found by studies that the ratio of d20:1-ganglioside to d18:1-ganglioside in the hippocampal gray matter of patients with AD is essentially dropped (Hirano-Sakamaki et al., 2015). In the entire hippocampus encompassing both white and gray matters, the proportion of GM1 (d20:1/C18:0) to GM1 (d18:1/C18:0) is not significantly different between controls and patients with AD. However, in patients with AD, the ratio of GM1 (d20:1/C18:0) to GM1 (d18:1/C18:0) in the outer molecular layer (ML) of the dentate gyrus is decreased. These studies indicate that the influence of the spatial arrangement of GM1 should be considered when investigating its role in the pathogenesis of AD. When studying the composition or ratios of these molecules, their distribution should be considered. The spatial arrangement of GM1 may also mediate the pathogenesis of AD. The lacking co-localization of gangliosides in the brain indicates that it is important to consider the spatial distribution of gangliosides when studying the mechanisms of ganglioside-mediated diseases.

## The Relationship Between LacCer and PD

Parkinson’s disease (PD) is a common neurodegenerative disorder, and its burden is expected to significantly increase with the aging of the global population (Connolly and Lang, 2014). The main pathological feature is the accumulation of intracellular α-synuclein in so-called Lewy bodies. However, dysregulation of lipid metabolism may also play an important role in the pathogenesis of PD (Bras et al., 2008). Studies have confirmed that the gene encoding glucocerebroside glycosidase (GBA) is the most common genetic risk factor for sporadic PD, accounting for about 7% of cases (Sidransky et al., 2009; Pchelina et al., 2017; Arkadir et al., 2018). It may also suggest a key role for glucosylceramide and ceramide metabolism in the development of PD and subsequent cognitive impairment. In a study on the lipid profile of idiopathic PD, there was a significant difference in GM3 ganglioside between patients with PD and controls, whereby higher plasma GM3 levels were associated with PD (Chan et al., 2017). Di Pasquale et al. (2010) identified a ganglioside-binding domain (GBD) in α-synuclein that has a clear preference for GM3. When Grey et al. (2015) isolated exosomes from neuroblastoma cells, they observed that GM3 and GM1 accelerated the aggregation of α-synuclein while other phospholipids slowed it down. It was reported that patients with PD had higher levels of hexosylceramide and lactosylceramide than controls, whereby the highest levels were observed in patients with PD with cognitive impairment (MCI or dementia) (Mielke et al., 2013). These results suggest that plasma lipid levels may be a potential predictor of PD risk and cognitive impairment. However, the small sample size and the lack of broader technical validation of lipid types and levels limits the strength of these findings, which should be validated in longitudinal follow-up studies. Aging is a significant risk factor for hereditary and sporadic PD (Rocha et al., 2015), and a study found elevated ceramide and GM1a levels in the brains of older rats (Hallett et al., 2018). Hallett et al. (2018) found that the ceramide disorder may speed up the pathophysiology of PD and age-related neurodegeneration. When the mouse Galgt1 gene (encoding GM2/GD2 synthase) was knocked out, mice showed dyskinesias similar to PD and decreased abundance of dopaminergic (DA) neurons in the substantia nigra (Wu et al., 2011). Moreover, the GM1 analog LIGA-20 relieved the symptoms of the mice, and the level of dopamine was restored to a certain extent. Therefore, GM1 may reduce α-synuclein aggregation by alleviating Ca^2+^ disorders in DA neurons (Ledeen and Wu, 2018). Studies have found that the level of glycosphingolipids in the mouse brain increases with age, such as GlcCer, LacCer, and GM1a, while neuraminidase activity in the body increases and lysosomal capacity is impaired (Hallett et al., 2018). The age-related increase in glycosphingolipids and abnormal protein degradation pathways can help explain the pathogenesis of PD, and the underlying mechanisms should be investigated in future studies.

## The Relationship Between LacCer and ALS

The characteristic manifestation of ALS is the loss of motor neurons, leading to dyskinesias. In an early study (Rapport, 1990), 17 of 21 patients with ALS were found to have abnormal ganglioside distribution. Except for the motor cortex, abnormal gangliosides were detected in the frontal lobe, temporal lobe, and parahippocampal cortex, which indicates that gangliosides are related to the underlying pathological mechanism of ALS. Gangliosides may affect the activity of the central growth factor or neurotrophic factor and interfere with the sensitivity of specific neurons to these factors. This change in neurons promotes pathological changes in ALS. At the same time, there are also reports that anti-ganglioside antibodies were found in the serum of patients with ALS (Mizutani et al., 2003). However, how gangliosides mediate the pathological process of ALS in the body is still unclear. In spite of ongoing studies, the correlation between gangliosides and ALS is still controversial. A recent study revisiting the role of ganglioside antibodies (Kollewe et al., 2015) found that although the levels of ganglioside antibodies were increased in the serum of some patients with ALS, in others it was comparable with normal controls. In contrast, there was no significant difference in the frequency of ganglioside antibodies. Although many scholars have speculated that ganglioside antibodies may be related to autoimmunity (Niebroj-Dobosz et al., 1999), it is regrettable that the existing evidence is insufficient to prove that the emergence of ganglioside antibodies is related to ALS. It was reported that the injection of gangliosides in patients with ALS does not alleviate the symptoms (Lacomblez et al., 1989), raising questions surrounding the connection of gangliosides with ALS.

However, animal and cell culture studies provide new evidence that gangliosides might have some involvement in the pathological process of ALS. The link shared by ganglioside antibody type, titer level, and ALS remains controversial. Nevertheless, in the case of the synthesis of glucosylceramide upstream of the ganglioside synthesis pathway being blocked, it will cause metabonomic changes in mice; influencing muscle function and movement endplate shape. The levels of GlcCer and downstream GM3 and GM2 gangliosides in SOD1 (G86R) mice (transgenic ALS model mice) were significantly higher than in wild-type littermates, and immunostaining results showed increased levels of gangliosides mainly located in the boundaries of muscle fibers (Henriques et al., 2015). It is possible that inclusions containing gangliosides cause denervation of muscle tissue and mediate the pathological process of ALS. GlcCer inhibitors have different effects on transgenic mice depending on the site of action. When GlcCer inhibitors directly act on the CNS, the survival time of transgenic mice is significantly shortened, but when GlcCer inhibitors act on the periphery, they can promote motor neuron regeneration and improve motor function. A recent study (Henriques et al., 2017) indicated that inhibition of the GlcCer-degrading enzyme β-glucocerebrosidase (GCase) improves the distribution of gangliosides in the body, delays the onset of the disease, and improves exercise function and retention of motor neurons. Furthermore, inhibition of GCase also accelerates the recovery of peripheral neuron function. Therefore, regulating the metabolism of glucosylceramide upstream of ganglioside may be an essential target for ALS treatment and drug development.

Lipid rafts play an important role in the development of diseases. The close relationship between ALS and cholesterol esters, another significant component of lipid rafts, has also been reported. For example, lack of cholesterol esters leads to the redistribution of NMDA receptor membranes in the body. Compared with type mice, there are significant differences in the protein composition of lipid rafts in G93A transgenic mice (Antonini et al., 2018). The analysis of structural changes in lipid rafts in ALS may open up new ideas for its treatment. Another study also pointed out that there is also a correlation between gangliosides in lipid rafts and ALS (Xu et al., 2015). In ALS transgenic mice, gangliosides GD1a and GT1b constitute as significant components of lipid rafts, and recombinant natural human IgM (rHIgM12) binds to it to increase α-tubulin tyrosination levels in the microtubule region, hence, stabilizing the microtubule structure and decreasing neuron and axon degeneration.

## Conclusion and Prospects

With the increasing life expectancy and aging of the global population, the number of patients with neurodegenerative diseases is continuously increasing (Feigin et al., 2017; GBD 2015 Neurological Disorders Collaborator Group, 2017). Unlike other diseases, neurodegeneration affects the cognitive function and motor skills of patients, leading to loss of basic self-care ability. This necessitates long-term support for basic everyday life, which is a great burden for family members and society.

Lactosylceramide is an early biomarker of neurodegenerative diseases. The development of sphingolipid analysis technology has made it possible to study the relationship between individual sphingolipids and illnesses. It was reported that high plasma levels of ceramide can predict hippocampal volume loss within a year (Mielke et al., 2010), and that ceramide may become an essential biomarker for predicting the progression of AD. A subsequent study (Mielke et al., 2012) confirmed the correlation between high levels of ceramide and the occurrence of AD. In a recent study, the plasma ratio of ultra-long-chain to long-chain ceramide was found to be correlated with the risk of AD. The study found that the plasma ratio is inversely proportional to the risk of AD. Therefore, the ratio of circulating ceramide can also be a potential diagnostic indicator for AD. In addition to ceramide, LacCer can also be used for the early diagnosis of diseases. The difference in serum sphingolipid concentrations can be used to diagnose inflammatory bowel disease. Among children with inflammatory bowel disease, the difference in the concentration of LacCer is the most significant compared with healthy children (Filimoniuk et al., 2020). Therefore, the level of LacCer may be closely related to the occurrence of inflammatory bowel disease, and it can be used as a biological marker for the diagnosis of inflammatory bowel disease in children. Similarly, LacCer synthase was found to have diagnostic specificity in patients with rectal cancer (Chatterjee et al., 2019). Targeting LacCer synthase may become an essential means for the treatment of rectal cancer in the future. The involvement of LacCer in diagnosing neurodegenerative diseases will contribute to their early treatment and early intervention. Age is the leading risk factor for most neurodegenerative diseases. A study on mice (Hallett et al., 2018) indicated that the levels of GSL, LacCer, GM1a, and other gangliosides in the brain increase with age, while the increase in LacCer levels will interfere with the functional stability of lysosomes, mitochondria, and other organelles. It also impacts the stability of normal biological functions in the body. Therefore, LacCer and gangliosides may be valuable diagnostic biomarkers and therapeutic targets for age-related neurodegenerative diseases.

The imbalance in cellular energy metabolism has always been closely related to certain neurodegenerative diseases. Cellular energy imbalance involves the dysfunction of organelles involved in regulating energy metabolism and the abnormal production and degradation of metabolites such as protein and lipids. Neuroinflammation is a significant cause of degenerative diseases of the CNS. The activation of the immune system of the body will increase energy consumption (Straub, 2012), and the increase in energy metabolism requirements of the body will lead to disorder in mitochondrial function, such as transformation of metabolism from aerobic oxidation to anaerobic glycolysis and increased release of free radicals, leading to oxidative stress (Gajewski et al., 2017). From a pathological point of view, a class of protein-related neurodegenerative diseases, such as ALS, AD, and PD, is caused by misfolding and changes in the process of protein synthesis, as well as blockages in the degradation pathway of the endoplasmic reticulum, leading to abnormal accumulation of toxic proteins and ultimately neurodegeneration. Although LacCer is synthesized in the Golgi apparatus, the formation of ceramide, which is the precursor of LacCer, is completed in the endoplasmic reticulum. Notably, abnormal dynamics of protein synthesis and degradation in the endoplasmic reticulum can also interfere with lipid synthesis. Ceramide is believed to contribute to the formation of stable membrane channels (Siskind and Colombini, 2000). The mitochondrial outer membrane is rich in ceramide, and it is speculated that ceramide may aid in the formation of protein channels in the mitochondrial outer membrane, thereby facilitating the release of pro-apoptotic proteins from mitochondria (Spincemaille et al., 2014). In addition, ceramide can damage the integrity of the mitochondrial electron transport chain (Di Paola et al., 2000), leading to an imbalance in mitochondrial dynamics (Spincemaille et al., 2014) and interfering with the regulation of cytochrome c (Aufschnaiter et al., 2017). Therefore, abnormalities in protein and lipid metabolism can lead to neurodegeneration, but the critical role played by the endoplasmic reticulum requires further study in the future.

The precise detection of LacCer is helpful for disease diagnosis and research. A recent study (Filimoniuk et al., 2020) showed that the serum sphingolipid profile of patients with inflammatory bowel disease is different from that of healthy patients. Among them, the level of LacCer in the serum of children with Crohn’s disease is significantly high. Consequently, the level of LacCer in serum is considered a potential biomarker for diagnosing Crohn’s disease. Therefore, the accurate identification and quantitative analysis of sphingolipid species can help clarify their relationship with neurodegenerative diseases and their role in disease progression. Commonly used classical analysis methods include thin layer chromatography (TLC), high performance liquid chromatography (HPLC), immunohistochemical methods, and pulse-chase experiments (Haynes et al., 2009). Liquid-chromatography-tandem mass spectrometry (LC-MS/MS) technology, which is HPLC and tandem mass spectrometry (MS/MS), can be used to detect structural changes in LacCer molecular species due to oxidative stress (Couto et al., 2015). Oxidative stress induced by inflammatory response is closely related to the development of neurodegenerative diseases. Therefore, LC-MS/MS and other technologies can help us study the correlation between LacCer and the occurrence and progression of neurodegenerative diseases. LacCer and its oxidative decomposition products are also potential new biomarkers for the clinical diagnosis of neurodegenerative diseases.

Due to lack of effective drug, neurological diseases are characterized by a progressive, irreversible pathological process. Resulting mental health problems and social burden are becoming the focus of individuals, families, and the government. The research on neurodegenerative diseases is deepening, and related research progress provides a theoretical basis for a better understanding of the disease, diagnosis, and drug development. The role of lipid metabolism disorder in associated diseases has also become a new research hotspot. The mechanisms of LacCer-mediated neurodegenerative diseases are diverse. LacCer can promote the activation of immune cells in the CNS, and it can be further degraded into ganglioside. In neurodegenerative diseases, the aggregation of misfolded proteins is aggravated and accelerates neurodegeneration. Gangliosides are an important component of lipid rafts, which play important roles in signal transduction, material transport, and energy metabolism. Overall, clinical research and basic experiments have confirmed the close relationship between LacCer and neurodegenerative diseases, which has opened up new ideas and means for detecting disease progression, innovation in therapeutic methods, and the development of targeted drugs. In future studies, it is hoped that a more in-depth understanding of the pathogenic mechanism of LacCer will open up broad prospects for the treatment of neurodegenerative diseases.

## Author Contributions

All authors listed have made a substantial, direct and intellectual contribution to the work, and approved it for publication.

## Conflict of Interest

The authors declare that the research was conducted in the absence of any commercial or financial relationships that could be construed as a potential conflict of interest.
